# Exploring the predictive value of combined ultrasound parameters for spinal anesthesia-induced hypotension in cesarean section: a prospective observational study

**DOI:** 10.1186/s12871-023-02160-7

**Published:** 2023-07-28

**Authors:** Shimiao Feng, Juan Gu, Chao Yu, Jin Liu, Juan Ni

**Affiliations:** 1 Department of Anesthesiology and Key Laboratory of Birth Defects and Related Diseases of Women and Children (Sichuan University), Ministry of Education, West China Second University Hospital, Sichuan University, No. 20, Section 3, Renmin South Road, Chengdu, 610000 China; 2 Department of Anesthesiology and Translational Neuroscience Centre, West China Hospital, Sichuan University, No. 37, Guo Xue Xiang, Chengdu, Sichuan 610041 China

**Keywords:** Hypotension, Cesarean section, Ultrasound, Velocity time integral, Left ventricular end-diastolic area

## Abstract

**Background:**

Prophylactic vasopressor infusion can effectively assist with fluid loading to prevent spinal anesthesia-induced hypotension. However, the ideal dose varies widely among individuals. We hypothesized that hypotension-susceptible patients requiring cesarean section (C-section) could be identified using combined ultrasound parameters to enable differentiated prophylactic medical interventions.

**Methods:**

This prospective observational trial was carried out within a regional center hospital for women and children in Sichuan Province, China. Singleton pregnant women undergoing combined spinal-epidural anesthesia for elective C-sections were eligible. Women with contraindications to spinal anesthesia or medical comorbidities were excluded. Velocity time integral (VTI) and left ventricular end-diastolic area (LVEDA) in the supine and left lateral positions were measured on ultrasound before anesthesia. Stroke volume, cardiac output, and the percentage change (%) in each parameter between two positions were calculated. Vital signs and demographic data were recorded. Spinal anesthesia-induced hypotension was defined as a mean arterial pressure decrease of > 20% from baseline. The area under the receiver operating characteristic curve (AUROC) was used to analyze the associations of ultrasound measurements, vital signs, and demographic characteristics with spinal anesthesia-induced hypotension. This exploratory study did not have a predefined outcome; however, various parameter combinations were compared using the AUROC to determine which combined parameters had better predictive values.

**Results:**

Patients were divided into the normotension (*n* = 31) and hypotension groups (*n* = 57). A combination of heart rate (HR), LVEDA_s_, and VTI% was significantly better at predicting hypotension than was HR (AUROC 0.827 vs. 0.707, *P* = 0.020) or LVEDA_s_ (AUROC 0.827 vs. 0.711, *P* = 0.039) alone, but not significantly better than VTI% alone (AUROC 0.827 vs. 0.766, *P* = 0.098).

**Conclusion:**

The combined parameters of HR and LVEDA_s_ with VTI% may predict spinal anesthesia-induced hypotension more precisely than the single parameters. Future research is necessary to determine whether this knowledge improves maternal and neonatal outcomes.

**Trial registration:**

ChiCTR1900025191.

## Background

In cesarean section (C-section) with spinal anesthesia, hypotension is a common side effect that can cause both maternal and fetal/neonatal adverse effects [1, 2]. The recommended prophylactic methods for spinal anesthesia-induced hypotension include vasopressor infusion, fluid loading, and left lateral tilting of the patient [3]. However, the ideal dose of vasopressor or fluid varies widely among individuals, as evidenced by the fact that hypotension does not occur in some patients even without vasopressor infusion and fluid loading [1, 4, 5]. Overtreatment with vasopressor infusion and fluid loading can increase the risk of other circulatory adverse events, such as hypertension, bradycardia, tachycardia, arrhythmia, and pulmonary edema [2–6]. Identifying patients at risk for hypotension can enable individualization differentiation of prophylactic interventions and protect patients from overtreatment.

However, no single predictor for spinal anesthesia-induced hypotension is widely accepted in clinical settings in terms of predictive value [3]. Our previous systematic review forecasted better predictive value of composite and dynamic parameters during a supine stress test (SST) [7]. An SST is defined as an assessment of the cardiovascular changes that occur due to inferior vena cava compression by the gravid uterus in the supine position, characterized by an increase in heart rate (HR) of > 10 bpm and a decrease in systolic blood pressure of > 15 mmHg. Point-of-care ultrasound is a convenient, fast, and non-invasive diagnostic method that can estimate and detect a patient’s blood volume status, cardiac output, and systemic vascular resistance, which are the components of blood pressure generation [8, 9]. Compared to a single predictor, we hypothesized that a combination of ultrasound parameters during the SST could predict hypotension in a C-section more precisely.

## Methods

This prospective observational study was conducted at the West China Second University Hospital. This study was approved by the Ethics Committee of West China Second University Hospital, Sichuan, China (Chairperson Prof Yi L. Xing) on 21/05/2019 (approval number: 2019039) and was registered at www.chictr.org.cn on 16/08/2019 (registration number: ChiCTR1900025191) as the first part of a multipart study. This article adheres to the STARD statement. We obtained written informed consent from all study participants before enrolment.

Singleton pregnant women with a gestational age of ≥ 37 weeks, 150–180 cm in height, undergoing combined spinal-epidural anesthesia (CSEA) for elective C-section were included in this study. Women were excluded if they had a contraindication to spinal anesthesia (including coagulopathies, allergies to local anesthetics, or sepsis) and medical comorbidities (including cardiac disease, preeclampsia, or a neurological disorder). Patients were also excluded if “successful spinal anesthesia,” defined as feeling no pain at the level of T6 within 10 min, was not achieved and if their ultrasound images were unclear (image grade ≤ 2). The image quality was graded on a categorical scale as previously described (1 = no image, 2 = poor and unusable image, 3 = usable image quality, 4 = good image quality, and 5 = perfect image quality) [9].

### Ultrasound measurements

Ultrasound measurements were performed by Dr Feng, who has an ultrasound operation qualification certificated by the National Health and Family Planning Commission (NHFPC) of China (Certification Number: 2018151510437, accessible at https://www.21wecan.com/).Before this, Dr Feng had been trained in transthoracic echocardiography (TTE) and transesophageal echocardiography for three months at the West China University Hospital and had TTE scanning experience of over 100 patients in one year.

After entering the operating room, the patients’ condition and baseline vital signs were monitored, and the baseline vital signs were recorded. Thereafter, Dr Feng performed TTE ultrasound measurements while patients were in the supine and left lateral positions. The patients rested for 2 min in each position before the ultrasound measurements were completed in 3 min. The same ultrasound device, a Philips CX50 (Philips, Bothell, Washington, USA) with a cardiac probe S5-1 (1–5 MHz), was used for all measurements. Anesthesia was administered immediately after the ultrasound measurements.

The velocity time integral (VTI) of subaortic flow was measured in the apical five-chamber view using pulsed wave Doppler with a 5-mm sample gate size in the left ventricular outflow chamber and by tracing the signal. Mean values of three consecutive VTI were recorded and marked as VTI_s_ for the supine position and VTI_l_ for the left lateral position. The left ventricular end-diastolic area (LVEDA) was measured once in each position on the parasternal short-axis at the level of the papillary muscles by tracing the blood-pool area at the end of the diastolic period. It was labelled as LVEDA_s_ and LVEDA_l_ for the supine and lateral positions, respectively. The left ventricular outflow tract diameter (D_LVOT_) was measured once on the parasternal long axis in the supine position at mid-systole, parallel to the aortic valve plane, within 0.5–1.0 cm of the valve orifice, inner-edge to inner-edge [10]. The left ventricular outflow tract cross-sectional area was calculated using the following equation: LVOT = π × (D_LVOT_/2)^2^.

### Calculations

We calculated the following parameters based on ultrasound measurement using the following formulae:

$$\mathrm{VTI\%}=({\mathrm{VTI}}_{1} -{\mathrm{VTI}}_{\mathrm{s}})/\mathrm{ VT}{\mathrm{I}}_{\mathrm{s}}$$

$$\mathrm{LVEDA\%}=({\mathrm{LVEDA}}_{1}-{\mathrm{LVEDA}}_{\mathrm{s}})/{\mathrm{LVEDA}}_{\mathrm{s}}$$

$$\mathrm{Stroke \ volume }(\mathrm{SV})=\mathrm{VTI}\times \pi \times (\mathrm{DLVOT}/2{)}^{2}$$

$$\mathrm{Cardiac \ output }(\mathrm{CO})=\mathrm{SV}\times \mathrm{HR}$$

$$\mathrm{Cardiac \ index }(\mathrm{CI})=\mathrm{CO}/\mathrm{body \ surface \ area}$$

$$\mathrm{Systemic \ vascular \ resistance }(\mathrm{SVR})=\mathrm{ Mean \ arterial \ pressure }(\mathrm{MAP})\times 80/\mathrm{CO}$$

$$\mathrm{Systemic \ vascular \ resistance \ index \ }(\mathrm{SVRI})=\mathrm{MAP}\times 80/\mathrm{CI}$$

### Anesthesia management

Pulse oximetry, non-invasive blood pressure, and a five-lead electrocardiogram monitoring was performed as routine perioperative patient monitoring (Philips IntelliVue; Philips Medical Systems, Andover, MA, USA). Patients were placed in the supine position for baseline vital signs measurement. MAP (mean arterial pressure) was measured at least three times at 1-min intervals. Baseline MAP was calculated as the average of three consecutive measurements with a variation rate of ≤ 10%. Spinal anesthesia-induced hypotension was defined as a decrease in the MAP of > 20% from baseline between spinal anesthesia induction and infant delivery. Lactated Ringer’s solution was infused at a dose of 8 mL/kg (maximum 500 mL) over 30 min from anesthesia, followed by a continuous infusion at a rate of 2 mL·kg^−1^·h^−1^. CSEA was performed with the patient in the left lateral position. An 18-gauge Tuohy needle was inserted into the L3–L4 intervertebral space, identified by the landmark of the iliac crest. The loss of resistance to saline technique was used to identify the epidural space. A 27-gauge Whitacre spinal needle was placed through the Tuohy needle until the dura mater was punctured. Plain 0.5% bupivacaine (2.5 mL for patient height < 165 cm or 2.7 mL for patient height ≥ 165 cm) was administered over 10 s for spinal anesthesia. An epidural catheter (Zhejiang Haisheng Medical Device Co., Ltd, Zhejiang, China) was inserted 4 cm into the epidural space. The patient was then returned to the supine position with 10° left lateral table tilt; the gradienter of an iPhone 7 Plus was used to measure the angle. Blood pressure and HR were measured at 1-min intervals. The operating table was returned to the horizontal position when the surgery started. A pinprick test was used to assess the anesthesia level at 4, 6, 8, and 10 min after termination of the spinal injection. No pain at the xiphoid level (T6) assessed at 10 min was defined as successful spinal anesthesia. Otherwise, an additional epidural of 2% lidocaine was titrated to effect, or ketamine was injected intravenously, and the patient was excluded from the study. Upper sensory block level was defined as no pain at dermatome level at 15 min after spinal injection. Phenylephrine 100 μg was injected intravenously when hypotension occurred and could be repeated to maintain MAP at ≥ 80% of that at baseline. Atropine 0.007 mg/kg was administered intravenously once up to a maximum dose of < 0.5 mg if the patient’s HR was ≤ 55 bpm. Other medical management provided was at the discretion of the attending anesthesiologist. An anesthesia resident recorded the vital signs. The attending anesthesiologist and anesthesia resident were required to stay away from the ultrasound machine to ensure that they were blinded to the ultrasound measurements. The ultrasound operator was not involved in the anesthetic management and was required to leave the operation room before anesthesia was commenced.

### Calculation of sample size

In our pilot study, the spinal anesthesia-induced hypotension rate was 62% (n = 13/21). The outcomes were the combination of parameters with a better predictive value for spinal anesthesia-induced hypotension by comparing the area under the receiver operating characteristic curve (AUROC) between parameter combinations. The sample size calculation in the study protocol was based on the assumption of an AUROC of 0.85, compared with an average AUROC of 0.7 in previous studies, using MedCalc 15.6.1 software (MedCalc Software, Ostend, Belgium) [11–13]. With α set at 0.05, β set at 0.20, and the power of the test (1 − β) set at 0.80, a minimum total of 81 cases, with 54 cases in the hypotension group and 27 cases in the normotension group, were needed to detect a statistically significant difference in the receiver operating characteristic (ROC) curve analysis.

### Statistical analysis

Data were presented as mean ± standard deviation, number (percentage), or median (interquartile range) as indicated. The normality of data was assessed using the Shapiro–Wilk test. The t-test, Fisher’s test, and Mann–Whitney U test were used to compare means, percentages, and medians. All these tests were two-sided.

SPSS 20.0 software (IBM Corp., Armonk, NY, USA) was used for logistic regression analysis and ROC curve generation. The inclusion of parameters in the multiple variable logistic regression was based on statistical and clinical considerations. For example, SV, CO, VTI%, SV%, and CO% are calculated based on VTI, and the parameter with the largest AUROC among these was included in multiple variable logistic regression. The selection of other data followed the same principle. The included parameters were analyzed using multiple variable logistic regression analysis to determine if they were independently associated with the incidence of spinal anesthesia-induced hypotension. Thereafter, the complex predictive index (CPI) of parameters was calculated using the following formula:

$$\mathrm{CPI}(\mathrm{y})=1/[1+{\mathrm{e}}^{-(\mathrm{x}1\times \mathrm{b}1+\mathrm{x}2\times \mathrm{b}2+...+\mathrm{xn}\times \mathrm{bn}+\mathrm{u})}]$$

ROC curves were used to test the ability of the identified parameters to predict hypotension, and the AUROC was calculated. AUROC ≤ 0.5 indicated no predictive ability, and AUROC = 1.0 indicated the best possible predictive ability. The maximal value of Youden’s index was used as the criterion for selecting the optimal cutoff values of the predictive parameters; Youden’s index was calculated as follows:

$$\mathrm Y\mathrm o\mathrm u\mathrm d\mathrm e\mathrm n'\mathrm s\;\mathrm i\mathrm n\mathrm d\mathrm e\mathrm x=\mathrm{sensitivity}+\mathrm{specificity}-1.$$

MedCalc 15.6.1 software (MedCalc Software, Ostend, Belgium) was used to test the difference in AUROC between parameters. For all analyses, *P* < 0.05 was considered statistically significant.

## Results

**Fig. 1:**
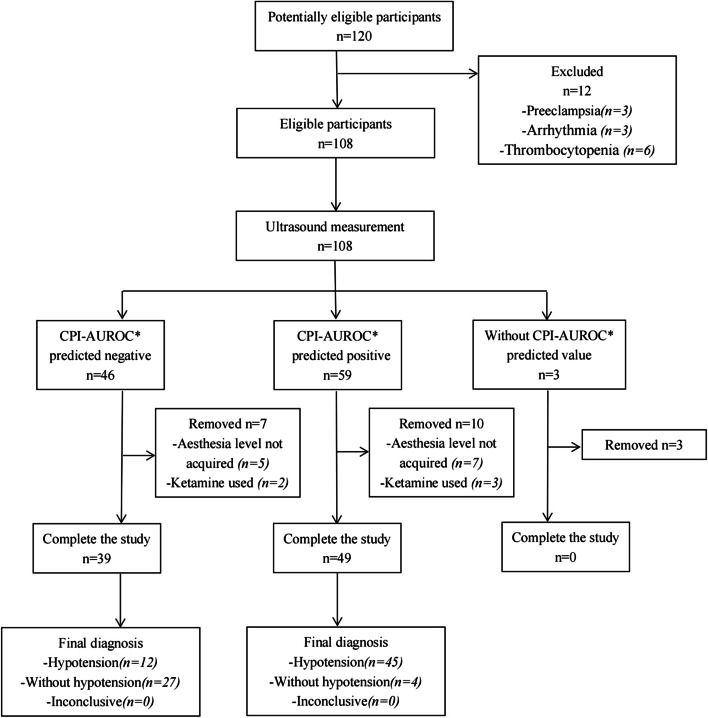
Flow chart of patient enrolment. *CPI-AUROC: area under the receiver operating characteristic curve of the complex predictive index

A total of 120 patients were evaluated for eligibility of inclusion in the study between August 2019 and February 2020. Twelve women were excluded due to pre-eclampsia, arrhythmia, or thrombocytopenia. Seventeen women were excluded after unsuccessful spinal anesthesia or administration of additional medications. Three women were excluded due to the inability to obtain clear ultrasound images. Ultimately, data from 88 women were included in the analyses (Fig. 1).

The demographic and anesthesia information for the participants are shown in Table 1. There were significant differences in baseline HR, phenylephrine administration, and maternal adverse reactions between the two groups (normotension group, *n* = 31; hypotension group, *n* = 57). No neonate had an Apgar score of < 7 at 10 min after delivery.

**Table 1:** Demographic characteristics and anesthesia information

	Normotension group (*n* = 31)	Hypotension group (*n* = 57)	*P* value
Age (years)	32.0 ± 4.7	32.9 ± 4.6	0.400
Height (cm)	160.4 ± 4.4	159.9 ± 4.8	0.659
Weight (kg)	67.9 ± 8.4	69.1 ± 7.6	0.491
Gestational age (d)	275 ± 9	274 ± 6	0.468
HR (bpm)^c^	77 ± 6	85 ± 12	< 0.001※
ΔHR (bpm)^a^	− 2 ± 5	− 3 ± 7	0.532
Upper sensory block level (dermatome)	T6 (T4–T6)	T6 (T4–T6)	0.761
Bupivacaine dose (mL)	2.5 (2.5–2.7)	2.5 (2.5–2.5)	0.412
Intravascular fluid volume (mL)	389 ± 119	400 ± 88	0.627
Time from anesthesia to surgery start (min)	16.9 ± 7.3	16.6 ± 4.6	0.778
Time from surgery start to delivery (min)	5.4 ± 2.8	5.8 ± 3.6	0.513
Average phenylephrine dosage per patient (µg)	0 (0–0)	200 (100–300)	< 0.001※
Patients requiring atropine [*n* (%)]	1 (3.2%)	4 (7.0%)	0.653
Maternal adverse reaction [*n* (%)]	1 (3.2%)	19^b^ (33.3%)	0.001※
Nausea (*n*)	0	5	
Vomiting (*n*)	0	0	
Shortness of breath (*n*)	1	12	
Dizziness (*n*)	0	3	

Values are presented as mean ± standard deviation or number (percentage)

^a^ΔHR = HR in left lateral position − HR in supine position

^b^One patient had both nausea and shortness of breath

^c^*HR* heart rate

※the outcome has statistically significant

Ultrasound measurements and the calculated parameters are presented in Table 2.

**Table 2:** Ultrasound measurements and the calculated parameters

	Normotension group (*n* = 31)	Hypotension group (*n* = 57)	*P* value
Parameters in supine position			
LVOT^a^ diameter (cm)	1.8 ± 0.1	1.8 ± 0.1	0.575
LVEDA_s_^b^ (cm^2^)	11.9 ± 2.7	10.4 ± 1.8	0.002※
VTI_s_^c^ (cm)	24.8 ± 3.6	21.7 ± 4.4	0.001※
SV_s_^d^ (ml)	63.8 ± 11.8	54.8 ± 12.8	0.002※
*CO*_s_^e^ (L·min^−1^)	4.9 ± 0.9	4.6 ± 1.0	0.227
SVR^f^ (dyn·s·cm^−5^)	1487 ± 295	1582 ± 390	0.243
SVRI^j ^(dyn·s·cm^−5^·m^−2^)	2536 ± 475	2711 ± 638	0.184
Parameters in left lateral position			
LVEDA_l_ (cm^2^)	12.4 ± 1.9	11.8 ± 2.0‡‡	0.217
VTI_l_ (cm)	22.8 ± 3.7‡‡	23.0 ± 4.0§§	0.798
SV_l_ (ml)	58.7 ± 11.0‡‡	58.2 ± 12.0§§	0.841
*CO*_l_ (L·min^−1^)	4.4 ± 0.8‡‡	4.7 ± 1.0	0.098
Percentage change between positions^¶¶^			
LVEDA%	7.0% ± 19.7%	16.2% ± 20.5%	0.044 ※
VTI%	− 7.4% ± 12.6%	8.3% ± 17.8%	< 0.001※
SV%	− 7.2% ± 12.7%	8.2% ± 17.8%	< 0.001 ※
*CO*%	− 9.3% ± 13.0%	4.3% ± 16.5%	< 0.001※

Values are presented as mean ± standard deviation

^a^*LVOT* left ventricular outflow tract, ^b^*LVEDA* left ventricular end-diastolic area, ^c^*VTI* velocity time integral, ^d^*SV* stroke volume, ^e^*CO* cardiac output, ^f^*SVR* systemic vascular resistance, ^j^*SVRI* systemic vascular resistance index

^‡‡^*P* < 0.01 compared to the supine position

^§§^ *P* < 0.05 compared to the supine position

^¶¶^Percentage change between positions: (value in the left lateral position − value in the supine position)/value in the supine position × 100%

※the outcome has statistically significant

In the supine position, there were significant differences in LVEDA_s_, VTI_s_, and SV_s_, but not in CO_s_, D_LVOT_, SVR, or SVRI between the two groups. There were significant differences in the percentage changes of LVEDA, VTI, SV, and CO between the supine and left lateral positions. However, all measurements in the left lateral position were comparable.

Single-parameter predictive values for spinal anesthesia-induced hypotension are listed in Table 3. SV, CO, VTI%, SV%, and CO% were calculated based on VTI; VTI% had the largest AUROC among these and was chosen as the representative parameter. Moreover, LVEDA_s_ was the best predictor among LVEDA_s_, LVEDA_l_, and LVEDA%, and HR was the only valuable predictor among vital signs and demographic data. Comprehensively, the top three parameters with the best predictive values (VTI%, LVEDA_s_, and baseline HR) were included in multiple variable logistic regression analysis. The logistic equation was as follows:

**Table 3:** Predictive values of parameters for spinal anesthesia-induced hypotension in cesarean delivery (*n* = 88)

	AUROC^e^	95% CI^f^	*P* value	Cutoff value	Sensitivity	Specificity
HR^a^	0.707	0.601–0.813	0.001	> 87	38.6%	100%
LVEDA_s_^b^	0.711	0.588–0.835	0.001	< 11.4	71.9%	67.7%
VTI_s_^c^	0.711	0.603–0.819	0.001	/	/	/
SV_s_^d^	0.711	0.596–0.825	0.001	/	/	/
LVEDA%	0.661	0.538–0.784	0.013	/	/	/
VTI%	0.766	0.665–0.868	< 0.001	> 1.3%	61.4%	87.1%
SV%	0.762	0.660–0.865	< 0.001	/	/	/
*CO*%^¶^	0.748	0.643–0.852	< 0.001	/	/	/
*HR* + *LVEDAs* + *VTI%*	0.827	0.732–0.900	< 0.001	> 0.638	78.9%	87.1%

^a^*HR* heart rate, ^b^*LVEDA* left ventricular end-diastolic area, ^c^*VTI* velocity time integral, ^d^*SV* stroke volume, ^¶^*CO* cardiac output, ^e^*AUROC* area under the receiver operating characteristic curve, ^f^*CI* confidence interval

$$\mathbf{C}\mathbf{P}\mathbf{I}=1/[1+{\mathbf{e}}^{-(\mathbf{V}\mathbf{T}\mathbf{I}\mathbf{\%}\times 5.648-0.231\times \mathbf{L}\mathbf{V}\mathbf{E}\mathbf{D}\mathbf{A}\mathrm{s}+0.059\times \mathbf{H}\mathbf{R}-1.606)}]$$

**Fig. 2:**
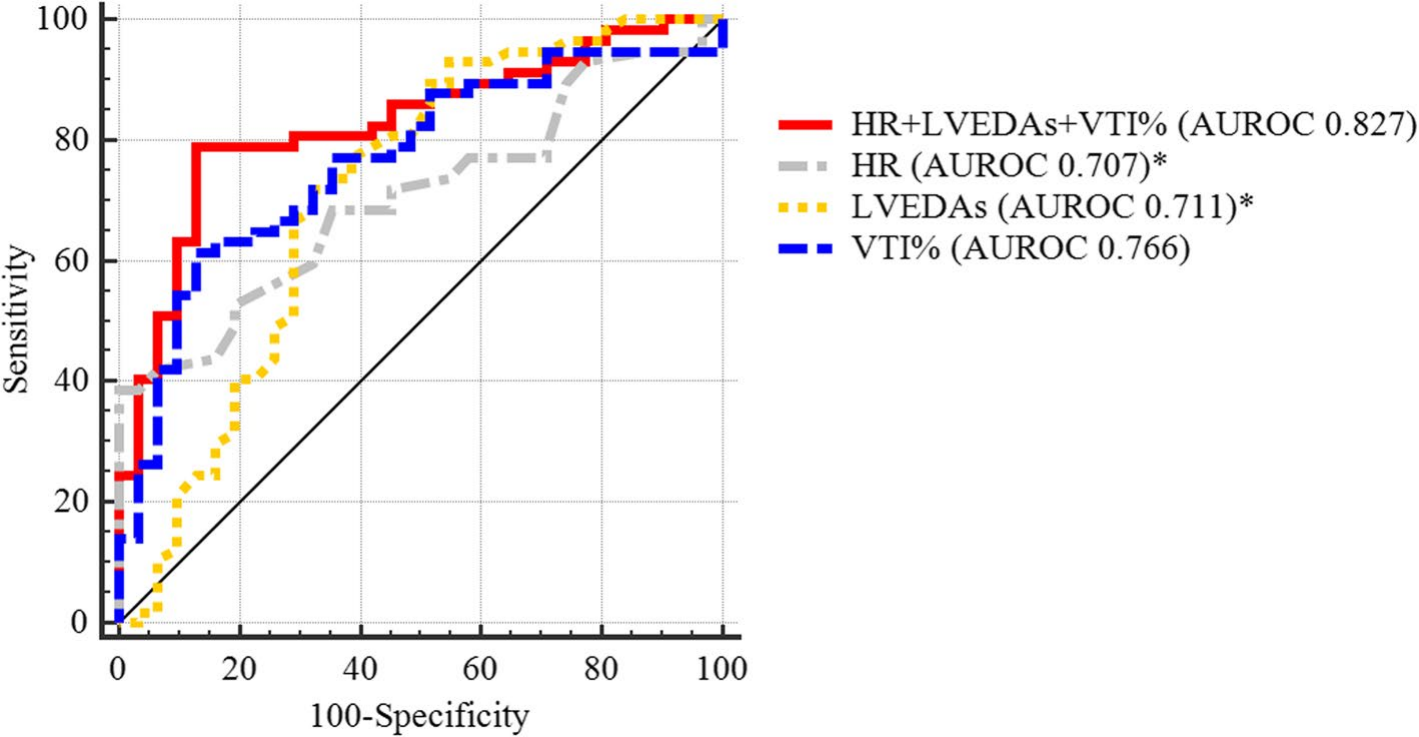
Area under the receiver operating characteristic curve of HR + LVEDA_s_ + VTI% compared to single parameter. HR: heart rate; LVEDA_s_: left ventricular end-diastolic area in supine position; VTI%: percentage change between positions of velocity time integral. *vs. HR + LVEDA_s_ + VTI%, *P* < 0.05

**Fig. 3:**
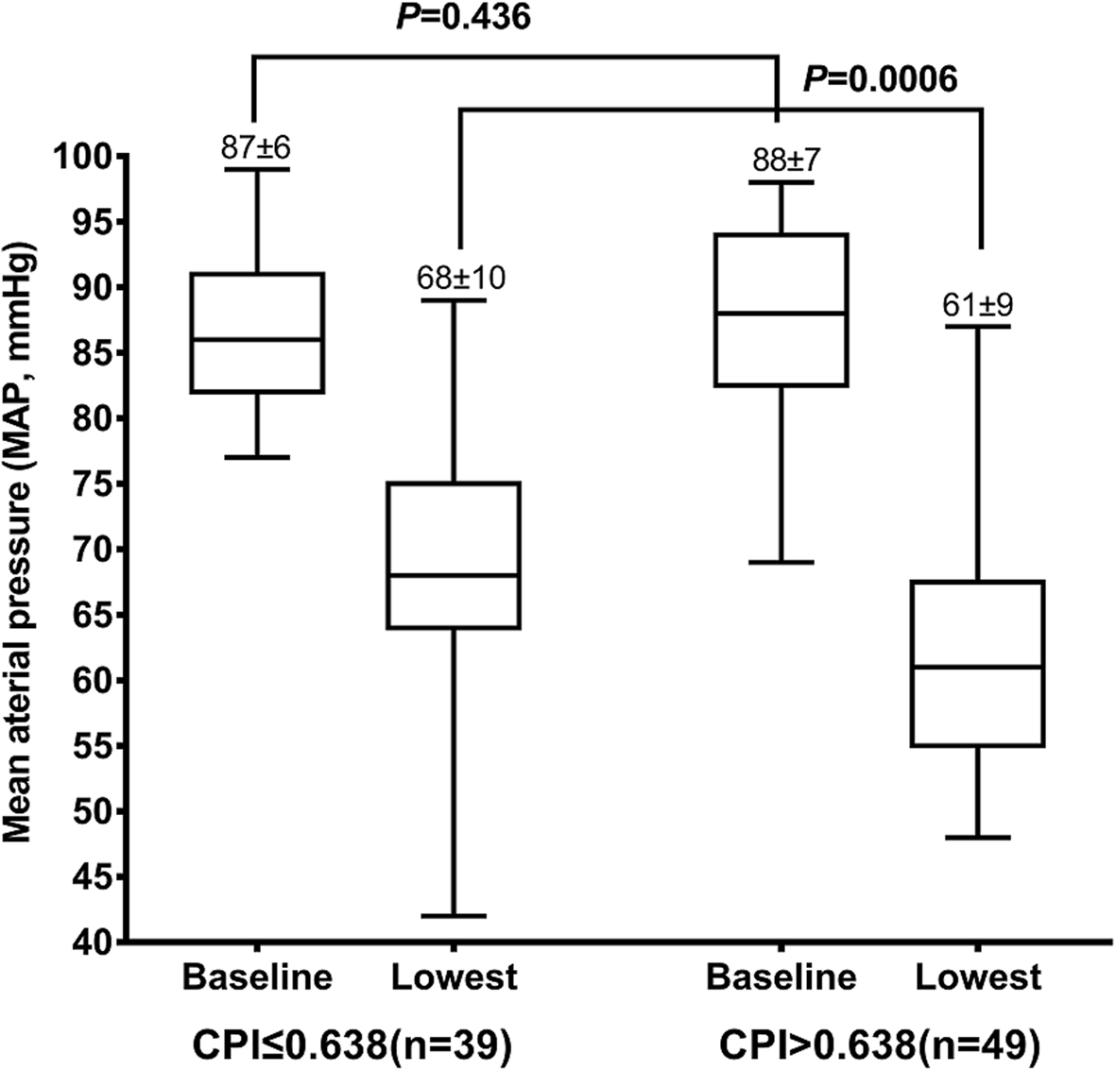
Mean arterial pressure change in patients with different CPI. *CPI: the complex predictive index

Furthermore, there was a statistically significant increase in the effectiveness and precision of the combination of HR, LVEDA_s_, and VTI% in predicting spinal anesthesia-induced hypotension (*P* < 0.001), compared to HR (AUROC 0.827 vs. 0.707, *P* = 0.020) and LVEDA_s_ (AUROC 0.827 vs. 0.711, *P* = 0.039) alone. The combination did not reach a statistically significant difference from VTI% (AUROC 0.827 vs. 0.766, *P* = 0.098; Fig. 2). When patients were divided into two groups according to the cutoff CPI value of (≤ 0.638 or > 0.638), patients with CPI > 0.638 had significantly lower MAP than those with CPI ≤ 0.638 (Fig. 3). The average decrease in MAP was approximately 20% in patients with CPI ≤ 0.638, while that in patients with CPI > 0.638 was approximately 30%.

## Discussion

The cause for spinal anesthesia-induced hypotension in C-sections is multifactorial [14, 15]. As the predictive value of combined predictors had not been studied before, we conducted this exploratory study to verify the possibility of further improving the predictive accuracy of spinal anesthesia-induced hypotension using a combination of multiple parameters. Baseline HR and LVEDA_s_ combined with VTI% may predict spinal anesthesia-induced hypotension in C-sections more precisely than a single parameter, as demonstrated by the study results. The CPI of the three parameters could effectively identify those patients who were more likely to develop hypotension.

Notably, HR, LVEDA_s_, and VTI% had independent predictive values as each plays a role in blood pressure generation [9]. However, the coefficients (b values) of the three parameters were 5.648, − 0.231, 0.059, respectively, in the CPI equation, which meant VTI% played a much more important role than HR and LVEDA_s_ in hypotension prediction. As a composite parameter of systolic cardiac function, blood volume, and CO, VTI% represented the dynamic change of VTI between the supine and left lateral positions and could predict spinal anesthesia-induced hypotension effectively in our study [16]. The outcome was quite similar to that reported by Zieleskiewicz and further proved the predictive value of VTI% [17]. As against Zieleskiewicz’s design, we measured VTI changes between the supine and left lateral positions to simulate aortocaval compression during the SST, as the SST was much easier and safer for participants than the passive leg raise test. Baroreceptor reflex sensitivity reflected the autonomic regulation of CO to maintain blood pressure, which was clearly diminished in the supine position during pregnancy [8, 18–20]. In short, VTI% was a valuable predictor for spinal anesthesia-induced hypotension during C-section. However, there were some problems when using the composite parameter to guide clinical treatment. Kim et al. used carotid artery-corrected flow time (FTc) for hypotension prediction with an excellent AUROC (0.922, 95% confidence interval 0.779 − 0.985, *P* < 0.001) [21]. FTc, as a composite parameter, is affected by the preload and afterload simultaneously. However, the authors mentioned the following in their discussion “…a low FTc cannot distinguish between low preload, high afterload, or both. Thus, between fluid therapy and vasopressors, it is not adequate to select a guide method to prevent or treat hypotension. If used in conjunction with an indicator that only detects the preload or afterload, this could be more clinically helpful” [21].

Therefore, LVEDA was also measured in our study as a standard ultrasound assessment of volume status [22]. The study confirmed no statistically significant difference in baseline volume status between normotensive or hypotensive parturients, as there was no difference in LVEDA_l_ between the two groups. However, LVEDA_s_ was more reduced than LVEDA_l_ in the hypotension group, possibly due to decreasing venous return due to aortocaval compression in the supine position, which could be a causative factor of hypotension. In a previous study, increasing baseline volume and CO was confirmed after fluid loading; however, these effects were not well maintained after spinal anesthesia and could not prevent hypotension effectively [23]. This could be the reason for its limited efficacy in preventing hypotension. Hence, we should reconsider the necessity of fluid loading and volume administration in C-sections to limit the risk of fluid overload and pulmonary edema [3, 6]. In addition to ultrasonic parameters, a baseline HR of > 87 bpm could also predict spinal anesthesia-induced hypotension with a sensitivity of 38.6% and a specificity of 100%. HR is usually faster during pregnancy due to increased sympathetic activity and for increasing the blood volume delivery to tissues within a given period of time [19]. However, HR compensation is limited; an excessively high HR may represent an insufficient cardiac reserve function or blood volume. Therefore, HR may be valuable as a predictor within a combination of predictors.

We investigated the predictive value of multiple parameters to find the most suitable predicting parameter and discovered that the predictive value of SV, CO, and their difference or variation rates were not better than that of VTI%. Moreover, CO was comparable between the groups in both the supine and left lateral positions, possibly due to HR compensation. These results suggest that the cardiovascular parameters during a period may not be as valuable as the immediate measured parameters in predicting spinal anesthesia-induced hypotension.

Diverse definitions of spinal anesthesia-induced hypotension have been used in previous studies [1]. Referring to Zieleskiewicz’s study, we used the definition of a decrease in MAP of > 20% from baseline to compare the predictive value of VTI% with the findings of their study [17]. Only one participant complained of shortness of breath in the normotension group; no fetal or neonatal depression occurred in both groups. Therefore, we inferred that, in the case of participants with no complications, maintaining the MAP ≥ 80% of that at baseline might be sufficient for adequate organ perfusion and preventing the adverse effects of hypotension in a C-section.

This study has some limitations. First, only the average lowest MAP of participants between the two groups was compared, but the time at which hypotension occurred and the duration were not compared. Although the time from anesthesia to delivery was comparable between the two groups, the duration varied from 12 to 52 min per participant, making statistical analysis difficult. Second, ultrasound measurement was time-consuming and required specific devices and skills, which would restrict the clinical application of the outcome. However, in our study, one certified operator with TTE scanning experience of > 100 patients in 1 year could guarantee the reproducibility of measurements. Point-of-care ultrasound evaluation is rapidly becoming incorporated into medical practice [10]. Both LVEDA and VTI measurements are basic applications of ultrasound evaluation and can be learned quickly. Third, the etiology of spinal anesthesia-induced hypotension is multifactorial, including the anesthesia level and the duration of the supine position [14, 15]. We attempted to keep spinal anesthesia equivalent between groups. Theoretically, the supine position was best avoided before delivery; the participants were returned to the supine position at the beginning of surgery in accordance with the practice of obstetricians in our hospital. The average time from the start of surgery to delivery of the infant was < 6 min and comparable between the normotension and hypotension groups. Fourth, the exact time interval between ultrasound measurement and surgery start was not recorded. However, anesthesia was performed immediately after the ultrasound measurements were completed. As the time interval from anesthesia to surgery start was comparable between the two groups, we inferred that the time interval from ultrasound measurement to surgery start was also comparable.

To conclude, HR and LVEDA_s_ combined with VTI% based on ultrasound measurement may predict spinal anesthesia-induced hypotension more precisely than a single parameter in patients undergoing C-sections. Furthermore, the predictive value of VTI% was further validated. These findings may be a valuable guide to implementing differential prophylactic interventions and further elucidate the causes of spinal anesthesia-induced hypotension in C-sections. However, these need to be validated in additional studies.

## Data Availability

The datasets used and analyzed during the current study are available from the first author upon reasonable request. fengshimiao123@163.com.

## Abbreviations

C-section: Cesarean section
VTI: Velocity time integral
LVEDA: Left ventricular end-diastolic area
AUROC: The area under the receiver operating characteristic curve
SST: Supine stress test
CSEA: Combined spinal-epidural anesthesia
NHFPC: National health and family planning commission
TTE: Transthoracic echocardiography
LVOT: Left ventricular outflow tract
SV: Stroke volume
CO: Cardiac output
CI: Cardiac index
SVR: Systemic vascular resistance
MAP: Mean arterial pressure
SVRI: Systemic vascular resistance index
CPI: Complex predictive index
